# Translation and Validation of the Arabic Version of the Eating Behavior After Bariatric Surgery (EBBS) Questionnaire

**DOI:** 10.1007/s11695-023-06480-y

**Published:** 2023-02-13

**Authors:** Nuha H. Alsehemi, Amal A. Alharbi, Rahaf S. Alamri, Bushra A. Fatani, Seham H. Alsenan, Iffat Elbarazi, Madhawi M. Aldhwayan

**Affiliations:** 1 Community Health Sciences, College of Applied Medical Sciences, King Saud University, Riyadh, 11433 Saudi Arabia; 2 Institute of Public Health, College of Medicine and Health Sciences, United Arab Emirates University, 15551 Al Ain, United Arab Emirates

**Keywords:** Obesity, Bariatric surgery, Arabic, Dietary recommendations, Lifestyle change, Compliance, Questionnaire, Translation, Validation, EBBS

## Abstract

**Purpose:**

Complications after metabolic and bariatric surgery are common due to the patient’s poor commitment to postoperative lifestyle changes. Therefore, intensive follow-up from a multidisciplinary team might improve outcomes. The present study aimed to translate and validate the Eating Behavior after Bariatric Surgery (EBBS) questionnaire into Arabic for use in clinical and research settings.

**Materials and Methods:**

The study followed World Health Organization guidelines for translation and questionnaire adaptation, including forward translation, back translation, pilot testing, and the creation of the final version of the tool. A total of 390 patients who had undergone metabolic and bariatric surgery 3 years ago or more were involved in testing the questionnaire’s validity and reliability.

**Results:**

The mean age of participants was 36 years (range: 20 to 70 years), 56% were females, 94.1% were Saudis, and 56% had bachelor’s degrees. The internal consistency of the questionnaire was tested using Cronbach’s alpha. One item (alcohol consumption) was excluded during the reliability analysis due to low variance. The reliability analysis results showed that the 10 items were internally consistent, with a Cronbach’s α of 0.851.

**Conclusion:**

The validation and reliability of the Arabic-language version of the EBBS questionnaire were found to be satisfactory. The presence of a validated Arabic version of this instrument may help practitioners estimate patients’ adherence to dietary and lifestyle recommendations after metabolic and bariatric surgery. Furthermore, the questionnaire may aid in identifying factors that influence the efficacy of these procedures.

**Graphical Abstract:**

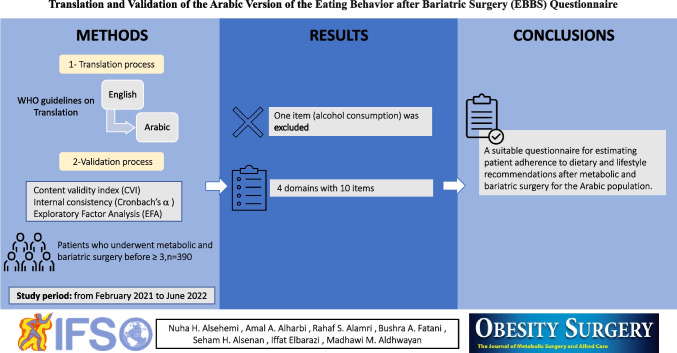

**Supplementary Information:**

The online version contains supplementary material available at 10.1007/s11695-023-06480-y.

## Introduction

Obesity is described by the World Health Organization (WHO) as “abnormal or excessive fat accumulation that presents a health risk.” Individuals with a body mass index (BMI) of 30 kg/m^2^ or higher are considered to have obesity [1]. In terms of prevalence, incidence, and economic burden, obesity poses a significant threat to public health at the national and global level [2]. Obesity has become a worldwide epidemic and is steadily increasing; according to the WHO, by 2025, the number of adults with obesity is expected to increase by more than one billion [3, 4]. Excess body fat associated with obesity results in chronic inflammation, leading to critical complications [5]. Consequently, the burden of disease associated with the high prevalence of obesity represents a significant global public health concern [6]. A variety of management approaches are available for treating obesity, including both surgical and non-surgical interventions [7]. Non-surgical treatments include a restricted diet, increased physical activity, pharmacology interventions, and behavioral changes [8]. For individuals whose lifestyle changes have failed, metabolic and bariatric surgery (MBS) is an alternative treatment option [9, 10]. MBS is a standardized treatment for obesity that produces long-term results by modifying the gastrointestinal anatomy to stimulate weight loss [11, 12]. Indications for undergoing MBS include a BMI ≥ 35 kg/m^2^ with or without the presence of obesity-associated medical problems, a BMI of 30–34.9 kg/m^2^ with metabolic disease, or a BMI ≥ 27.5 kg/m^2^ for the Asian population. With appropriate physician guidance and candidate criteria, MBS is a low-risk intervention for obesity and associated medical problems [13]. However, long-term complications can result when patients fail to follow the recommended lifestyle and dietary changes after surgery. These patients may be at high risk for decreased nutrient intake, nutrition deficiencies, and weight regain (WR) [14, 15]. WR is a common challenge after MBS when patients demonstrate poor commitment to lifestyle changes after surgery. Therefore, intensive follow-up by a multidisciplinary team might aid in determining the optimal course for a patient to follow. Such follow-up involves undergoing comprehensive nutritional evaluations of lifestyle changes, following a diet plan, engaging in physical activity, monitoring nutrition status and eating habits, pursuing weight loss maintenance, understanding the sensations of hunger and satiety, and assessing one’s quality of life. Follow-up empowers the patient to prevent poor outcome [16].

Nutritional assessment is an integral part of the patient’s journey post MBS. The use of simple, validated, and inexpensive tools to assess nutritional adherence post-surgery is crucial and helpful, especially in clinical settings. The Eating Behavior after Bariatric Surgery (EBBS) questionnaire was validated in the English language to evaluate the behavior and compliance of patients regarding dietary recommendations three or more years after undergoing MBS. The questionnaire consists of four domains: food (A), drinks (B), behaviors (C), and lifestyle (S). All domains were integrated to assess weight loss and compliance more efficiently after a patient underwent MBS [17].

Due to the increased number of metabolic and bariatric surgeries performed in the Middle East and North Africa (MENA) region [18], the present study aimed to translate and validate the EBBS questionnaire. The Arabic-language tool will be useful for assessing patients’ compliance with diet and lifestyle changes post MBS in clinical and research settings.

## Materials and Methods

The study was conducted to translate the EBBS questionnaire to the Arabic version using the WHO guidelines for translating, adapting, and validating an instrument [19]. As there was no existing Arabic version, permission was obtained from the primary authors of the original questionnaire; Dr. Daniele Santi approved the study [17]. The study was also approved by the Ethical Approval Committee of King Saud University, No. KSU-HE-21–444 on 16/09/2021.

### Study Design and Setup

This cross-sectional survey method study, performed from February 2021 to June 2022, included patients who underwent MBS 3 years ago or more. Consent was obtained from the participants before they completed the questionnaires.

### Translation

#### Forward and Back Translation

Five independent bilingual health professionals and two experts in patient nutrition following MBS translated the EBBS questionnaire from English to Arabic. All of the experts’ native language was Arabic, and they were fluent in English. The translation was based on achieving linguistic and conceptual equivalence with the original English version rather than producing a literal translation. After forward translation, an expert panel met with two specialists. One of them had experience with patient nutrition after MBS. The other was acquainted with instrument developments and was responsible for reconciling and resolving discrepancies between the two translated versions to produce a single translated version.

Next, a back translation was utilized to translate the Arabic version into English. The translation was sent to a bilingual expert in medical translation, whose native language was English and who had experience in the medical field, to check the accuracy of the initial Arabic version of the questionnaire against the original questionnaire. The translator was unaware of the original English version. Subsequently, the research group reviewed the back-translation and applied the necessary changes. Last, four Arabic-language specialists corrected the grammar and writing errors to finalize the questionnaire.

#### Pilot Testing of the Pre-final Version

During the cognitive debriefing testing of the pre-final version, twenty participants (10 males and 10 females) who underwent MBS 3 or more years ago completed the questionnaires via a phone interview after consenting to participate. While completing the questionnaires, participants were asked whether the translated questions and words were (a) clear and understandable, (b) difficult to answer, (c) confusing to answer, and (d) relevant to the topic. In addition, participants were asked to suggest any changes that might enhance the Arabic version. The interviewer also attempted to identify any thoughts or feelings associated with answering the questions and with the response categories. When applicable, suggestions were made to improve the questionnaire’s cultural adaptation, understanding, and clarity. For instance, volunteers suggested changing the wording in question 5 from “spirit drinks” to “alcoholic drinks.”

Additionally, in question 9, the participants did not fully comprehend how to determine the serving size of vegetables. We added clarification after the question (one serving of vegetables = half cup cooked vegetables or one cup raw vegetables) as well as in each answer (large = more than two servings; medium = two servings; small = less than two servings), according to guidelines [20].

To further determine the conceptual and content equivalence of the Arabic version, a second validation process was enacted to assess the content validity. This process involved 10 expert panel investigators (seven healthcare professionals, two public health professionals, and one Arabic-language specialist). Expert panel were contacted by email and invited to participate and assist with cultural adaptation and semantic evaluation of the questionnaire. After providing consent, experts received an online email questionnaire using Google Forms. The online survey contained all 11 questions translated into Arabic. The expert panel then assessed each item on the following scale for content equivalence (content-related validity), where 1 = not applicable, 2 = unable to determine relevance, 3 = relevant but requires minor revision, and 4 = very relevant and succinct for clarity, and made suggestions to improve the conceptual equivalency of the questionnaire when applicable. The responses obtained from the experts, along with semantic differentiation, were used to calculate the mean grades for the evaluation of clarity and content validation of each item. Based on the results of this process stage, the last version was edited into a final version, which was used in the next phase of the study.

#### Recruitment and Participants for Testing the Validity and Reliability of the Arabic Version of the EBBS Questionnaire

The sample size was calculated using the item–subject ratio. According to the recommendation, a sample size of 110 patients was required, with a ratio of 1:10 [21]. Based on the structural equation modeling technique for sample size calculation, 241 is the minimum sample size needed to conduct confirmatory factor analysis (CFA) [22]. To meet this standard, a minimum sample size of 351 participants was required for our analysis.

#### Questionnaire

**Fig. 1:**
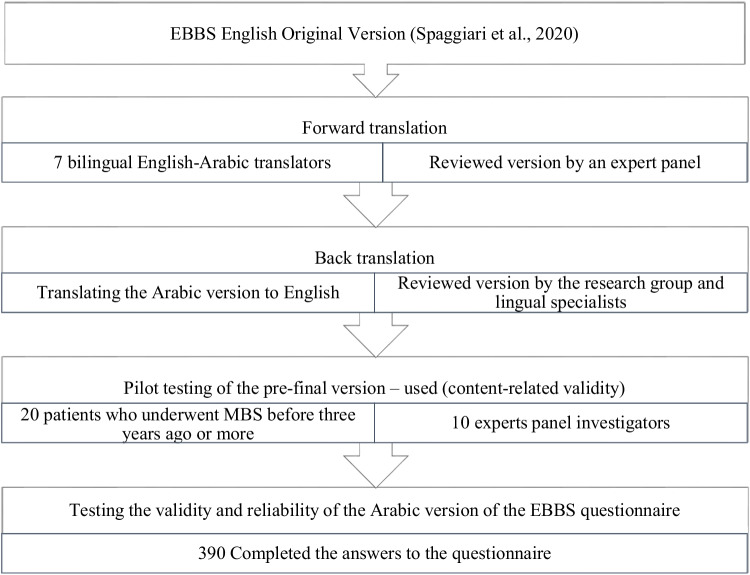
Process stages translation of the Arabic version of the Eating Behavior after Bariatric Surgery (EBBS) questionnaire

The final Arabic version of the EBBS questionnaire was uploaded to Google Forms and distributed via various social media platforms (Twitter, Telegram, WhatsApp, and Facebook). The first page of the survey was a consent form that would not allow the participant to start the next page unless they pressed the “Ok” button indicating their approval to join the study. Those who refused to participate were directed to a page thanking them for their time. In contrast, those who agreed were directed to the first page of the survey, which was divided into two parts. The first part assessed the sociodemographic characteristics of the participants (age, gender, educational level, country, and region). It also included the date of the participant’s operation and their body measurements in order to calculate weight-related outcomes (i.e., previous weight before the operation, nadir weight (the minimum weight that was reached after the operation), current weight, and height) [23]. The second part of the survey was the questionnaire, which consisted of 11 items divided into four functional domains: domain A (food), domain B (drinks), domain C (behaviors), and domain S (lifestyle). In the original questionnaire, all answers were assigned a score ranging from 0 to 2; the higher the final score, the better the patient’s adherence to the recommendations. Figure 1 summarizes the stages of the Arabic questionnaire translation process.

### Statistical Analysis

The mean ± standard deviation (SD) described continuous variables, and the categorically measured variables were described using frequencies and percentages. The histogram and the Kolmogorov–Smirnov test were applied to test the statistical normality assumption, and Levene’s test was used to assess the homogeneity of the statistical variance assumption. The EBBS questionnaire subscale scores were computed as item parcels by adding the subscale indicators yielding total subscale scores. The Cronbach’s α test was calculated for the questionnaire subscales to assess the internal consistency of the measured questionnaire. A Cronbach’s α of 0.70 to 0.80 is considered acceptable for a research scale, and an α greater than 0.80 is considered reliable [24]. The exploratory factor analysis (EFA) using principal components analysis (PCA) and parallel analysis (PA) tests were applied to the indicators measured using the EBBS eating behavior questionnaire [25]. Closeness-to-unidimensionality tests (UniCo, M-IREAL, and ECV) were used to assess the unidimensionality of the EBBS eating behavior questionnaire indicators and the overall subscale scores of the MBS participants [26]. The content validity index (I-CVI) and scale validity index (S-CVI) were used, with I-CVI of 0.78 or higher and S-CVA/Ave of 0.90 or higher representing the minimum acceptable indices [27]. Pearson’s correlation test was used to assess the correlations between the measured concepts [28]. Weight-related outcomes (participants’ percent total weight loss (%TWL) after MBS, percentage of weight loss maintenance (%WLM) after surgery, and WR) were dichotomized based on the mean scores of the two outcomes, categorizing them as (0 = low or 1 = high). The multivariate binary logistic regression analysis was used to determine which factors may contribute to MBS patients’ weight loss in the immediate post-surgical period and their odds of maintaining that weight loss [29]. In the multivariate logistic regression, the association between predictors and outcome was expressed using multivariate-adjusted odds ratios (OR) with 95% confidence intervals [30]. The SPSS IBM statistical analysis program was used for the data analysis. The FACTOR stand-alone statistical program [31] was used to perform the PA and closeness-to-unidimensionality tests in the EFA/PCA context. The statistical alpha significance level was set at 0.050.

## Results

### Descriptive Analysis

Table 1 shows the descriptive analysis for sample characteristics and anthropometric measurements. A total of 390 participants provided their responses. The mean age was 36.82 ± 9.67 years, more than half were females (56.2%), and (55.9%) had a bachelor’s degree. Moreover, the majority of participants (94.1%) were Saudis.

**Table 1:** The study sample’s sociodemographic characteristics (*N* = 390)

	Frequency (%)
Sex	
Male	171 (43.8)
Female	219 (56.2)
Age (years)	36.82 ± 9.67
Age group	
20–30 years	104 (26.7)
31–40 years	171 (43.8)
41–50 years	74 (19)
≥ 51 years	41 (10.5)
Educational level	
High school or less	74 (19)
Diploma degree	46 (11.8)
Bachelor’s degree	218 (55.9)
Higher studies	52 (13.3)
Place of residence	
KSA	367 (94.1)
Outside KSA	23 (5.9)
Time since undergoing metabolic and bariatric surgery	
3 years	121 (31)
4–6 years	169 (43.3)
7–10 years	63 (16.2)
≥ 11 years	37 (9.5)
BMI pre-surgery (kg/m^2^)	44.96 ± 7.66
BMI pre-surgery (kg/m^2^)	
Overweight	5 (1.3)
Obese class I	11 (2.8)
Obese class II and class III	374 (95.9)
BMI post-surgery (kg/m^2^)	28.71 ± 5.71
BMI post-surgery (kg/m^2^)	
Underweight	13 (3.3)
Normal	99 (25.4)
Overweight	135 (34.6)
Obese class I	91 (23.3)
Obese class II and class III	52 (13.3)
Weight-related outcomes	
Height (M)	1.66 ± 0.097
Pre-surgery weight (kg)	124.01 ± 27.33
Current weight (kg)	78.70 ± 17.23
Nadir weight (kg)	70.35 ± 15.16
Weight regain (kg)	8.34 ± 9.6
Total weight lost (kg)	45.33 ± 22.93
%TWL	35.44 ± 11.63
%EWL	87.30 ± 51.21
%WLM	84.10 ± 22.23

*KSA*, Kingdom of Saudi Arabia; *BMI*, body mass index; Mean ± SD; *%TWL*, percent total weight loss; *%EWL*, percent excess weight loss; *%WLM*, percent weight loss maintenance

**Fig. 2:**
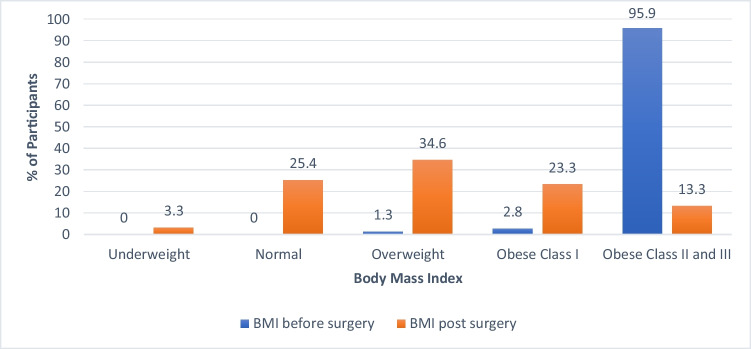
The participants’ BMI levels before and after metabolic and bariatric surgery

According to the findings, 31% of the participants had MBS 3 years ago. The pre-surgery average weight was 124.01 ± 2 7.33 kg, and the current post-surgery weight was 78.70 ± 17.23 kg. Furthermore, the nadir weight was 70.35 ± 15.16 kg, but the total weight loss was 45.33 ± 22.93 kg. On average, the %WLM was 84.10 ± 22.23, and the mean WR was 8.34 ± 9.6 kg. Figure 2 presents the participants’ BMIs before and after surgery.

### EBBS Questionnaire Adherence Information

Table 2 displays the results of the descriptive analysis of MBS participants’ perceptions of their EBBS questionnaire indicators.

**Table 2:** The EBBS questionnaire adherence information

Domain		Frequency (%)
Food consumption	Q1) How many meals do you eat per day?	
	3 main meals + 4–6 snacks	58 (14.9)
	3 times (breakfast, lunch, and dinner)	148 (37.9)
	3 main meals + 2–3 snacks	184 (47.2)
	Q4) How many times a week do you eat sweets? (Excluding breakfasts)	
	More than 3 times/week	271 (69.5)
	2 times/week	70 (17.9)
	0–1 time/week	49 (12.6)
	Q9) How is the portion of vegetables you consume during the meal?	
	Large	22 (5.6)
	Medium	121 (31)
	Small	247 (63.3)
Drink consumption	Q3) How much water do you drink during the meal?	
	More than 2 glasses	55 (14.1)
	1 glass	81 (20.8)
	None	254 (65.1)
	Q5) How many times a week do you drink wine or alcohol?	
	More than 2 times/week	10 (2.6)
	1 time/week	15 (3.8)
	Never	365 (93.6)
	Q6) How many times a week do you drink sparkling beverages?	
	More than 2 times/week	99 (25.4)
	1 time/week	95 (24.4)
	Never	196 (50.3)
General behaviors	Q2) How quickly do you consume the main meals?	
	10–15 min	188 (48.2)
	15–20 min	137 (35.1)
	More than 25 min	65 (16.7)
	Q7) How hungry do you feel before meals?	
	Elevated	94 (24.1)
	Medium	216 (55.4)
	Scant	80 (20.5)
	Q8) How full do you feel after meals?	
	Scant	3 (0.8)
	Medium	108 (27.7)
	Elevated	279 (71.5)
Lifestyle	Q10) How much time do you spend on physical activity?	
	0–1 time/week 30–60 min	191 (49)
	2–3 times/week 30–60 min	110 (28.2)
	5 times/week 30–60 min	89 (22.8)
	Q11) How often do you weigh yourself?	
	Never	78 (20)
	1 time/month	179 (45.9)
	1 time/week	133 (34.1)

#### Food Consumption

Approximately half of the participants (47.2%) consumed three main meals and two to three snacks per day, 69.5% consumed sweets more than three times per week, and 63.3% consumed an insufficient amount of vegetables with each meal.

#### Drink Consumption

Most participants (65.1%) did not drink water while eating. Furthermore, 93.6% of the patients had never consumed alcohol, and (50.3%) had never consumed sparkling beverages.

#### General Behaviors

Nearly half (48.2%) of participants consumed their food in less than 15 min, and (55.4%) rated their hunger level as a medium just before meals. The majority of participants (71.5%) reported an increase in post-mean satiety.

#### Lifestyle

Regarding the time spent in physical activity, 49% of the participants exercised 0–10 times for 30–60 min per week, and 45.9% weighed themselves once a month.

### Eating Behavior After Bariatric Surgery (EBBS) Score Distribution Among Domains

**Fig. 3:**
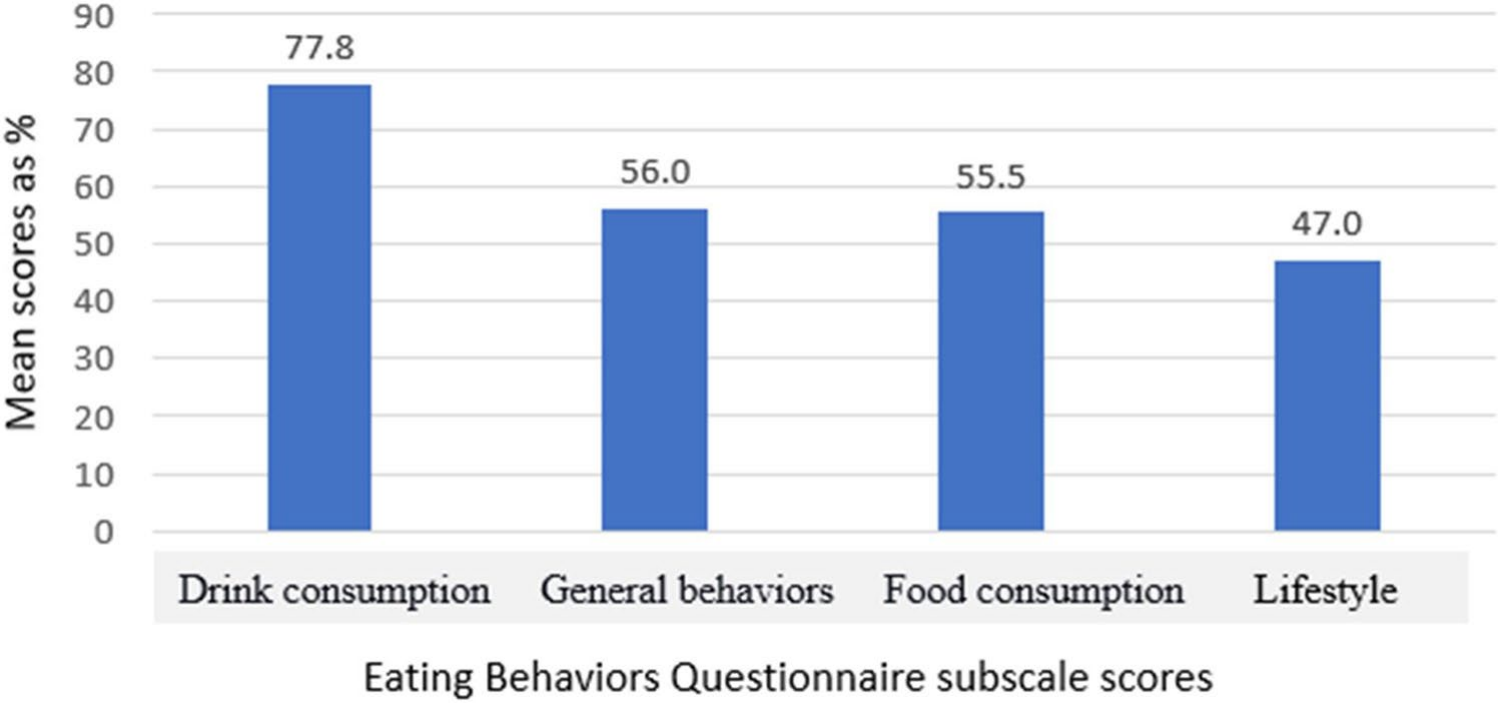
The participants’ compliance with desired eating behaviors expressed as percent mean scores

The descriptive analysis for the participants’ overall eating behavior subscale scores is shown in Table 3. The food consumption behaviors of bariatric participants mean scored 3.33 points, indicating low compliance with food consumption behaviors. The behaviors mean score was 3.36, indicating significant but low compliance with general behaviors. Conversely, the drink consumption behaviors mean score was 4.67, indicating high compliance with drinking behaviors. In contrast, the participants’ lifestyle mean score was 1.88 points, indicating poor compliance with lifestyle behaviors. Figure 3 illustrates the participants’ adherence to EBBS. Drink, behaviors, food, and lifestyle were assigned according to the highest score distribution of eating behaviors.

**Table 3:** Descriptive analysis of eating behaviors for participants post-surgery

Domain	Mean ± SD	Score possible range
Food consumption	3.33 ± 1.15	0–6 points
Drink consumption	4.67 ± 1.24	0–6 points
General behaviors	3.36 ± 1.13	0–6 points
Lifestyle	1.88 ± 1.14	0–4 points

### Questionnaire Reliability—Internal Consistency

One item (alcohol consumption) was excluded due to low variance. The reliability analysis indicated that the 10 items characterizing the bariatric participants eating behaviors and lifestyles were internally consistent, with a Cronbach’s α of 0.851. By applying the exploratory non-linear factor analysis, we found that all items loaded differently than their intended latent factors. Furthermore, the closeness-to-unidimensionality tests revealed that the 10 EBBS questionnaire indicators did not necessarily comprise a unidimensional trait (UniCo = 0.321 and ECV = 0.241).

### Content Validity

The mean grades for the evaluation of clarity and content validation of each item, along with semantic differentiation, were calculated using the responses obtained from the experts. The S-CVI values for all questions were between 1 and 0.9 except for question five, which was 0.6; the discrepancy was due to religion and social and cultural traditions (Supplementary 1).

### Correlation Between EBBS and Demographic Data

Correlation was determined by applying the bivariate Pearson’s correlations between the bariatric participants’ measured eating behavior scores and MBS-related anthropometric outcomes.

A negative correlation between compliance with drinking behaviors and pre-surgical weight (*r* =  − 0.19, *P* < 0.01) was shown. In contrast, food consumption, general behaviors, and lifestyle scores did not correlate with pre-surgical weight.

After MBS, participants’ BMI correlated negatively with their lifestyle score (*r* =  − 0.12, *P* < 0.05). Moreover, there was a positive correlation between compliance with food consumption behaviors (*r* = 0.12, *P* < 0.05), general behaviors (*r* = 0.12, *P* < 0.05), and lifestyle scores (*r* = 0.13, *P* < 0.05) and the participants’ TWL; however, compliance with drink consumption behaviors correlated negatively with the participants’ TWL (*r* =  − 0.11, *P* < 0.05).

The participants’ post-surgical %WLM was positively correlated with their compliance with lifestyle score (*r* = 0.18, *P* < 0.01). A negative correlation with WR scores was observed between compliance with drink consumption (*r* =  − 0.13, *P* < 0.01), general behavior (*r* =  − 0.10, *P* < 0.05), and lifestyle scores (*r* =  − 0.18, *P* < 0.01).

The scores indicating participants’ compliance with food and drink behaviors were positively correlated (*r* = 0.11, *P* < 0.05). Furthermore, compliance with drink consumption behaviors was positively correlated with lifestyle scores (*r* = 0.12, *P* < 0.05; see Table 4).

**Table 4:** Correlation between EBBS questionnaire and demographic data

	Pre-Wt	BMI-pre	Nadir weight	Post-Wt	Ht	BMI-Post	TWL	%TWL	%EWL	%WLM	WR	Food	Drink	General behaviors
Pre-Wt	1													
BMI-pre	.821^**^													
Nadir weight	.549^**^	.441^**^												
Current weight (Kg)	.550^**^	.421^**^	.832^**^											
Height score (Ht)	.577^**^	.037	.372^**^	.430^**^										
BMI-post	.253^**^	.449^**^	.676^**^	.828^**^	− .137^**^									
TWL	.779^**^	.662^**^	.029	− .096	.365^**^	− .321^**^								
%TWL	.433^**^	.389^**^	− .298^**^	− .467^**^	.189^**^	− .628^**^	.867^**^							
%EWL	− .195^**^	− .321^**^	− .366^**^	− .463^**^	.106^*^	− .575^**^	.116^*^	.277^**^						
%WLM	.094	.119^*^	.113^*^	− .353^**^	− .010	− .389^**^	.377^**^	.534^**^	.246^**^					
WR	.121^*^	.058	− .087	.481^**^	.184^**^	.417^**^	− .218^**^	− .368^**^	− .254^**^	− .810^**^				
Food consumption	.074	.104^*^	− .041	− .038	.004	− .026	.117^*^	.100^*^	.023	.009	− .003			
Drink consumption	− .188^**^	− .092	− .088	− .152^**^	− .177^**^	− .057	− .110^*^	− .015	− .005	.074	− .134^**^	.111^*^		
General behaviors	.070	.078	.015	− .043	− .010	− .049	.116^*^	.115^*^	− .019	.079	− .100^*^	.040	− .026	
Lifestyle	.039	.029	− .007	− .107^*^	.022	− .117^*^	.126^*^	.141^**^	.020	.179^**^	− .180^**^	.044	.124^*^	.097

*Pre-Wt*, pre-surgical weight; *BMI*, body mass index; *BMI*-*pre*, BMI pre-surgery; *BMI-post*, BMI post-surgery; *TWL*, total weight loss; *%TWL*, percent total weight loss; *%EWL*, percent excess weight loss; *%WLM*, percent weight loss maintenance; *WR*, weight regain; *HT*, height. **Correlation is significant at the 0.01 level (2-tailed). *Correlation is significant at the 0.05 level (2-tailed)

### Odds Ratios for EBBS Adherence Status Score and Demographic Variables

#### Percent Total Weight Loss (%TWL)

The descriptive analysis of the participants’ %TWL indicated that 52.3% had lost ≥ 35% of their weight post-surgery (Table 5).

**Table 5:** %TWL > 35.5% previous weight

	Frequency (%)
Loss weight < 35%	186 (47.7)
Loss weight ≥ 35%	208 (52.3)

Younger participants were 0.96 times more likely to lose weight post-surgery, *P* < 0.001 (Table 6). The gender of the participants correlated with weight loss post-surgery; males were five times more likely than females to lose weight post-MBS, *P* < 0.001.

**Table 6:** Multivariate logistic binary regression analysis of participants’ odds of %TWL ≥ 35% baseline weight in the post-surgical period

	Adjusted odds ratio (OR)	95% C.I		*P*-value
		Lower	Upper	
Age (years)	.955	.930	.980	< 0.001
Sex = male	4.559	2.803	7.414	< 0.001
Educational level	.835	.645	1.080	.170
Compliance with food consumption score	1.049	.861	1.278	.636
Compliance with drink consumption score	1.074	.886	1.302	.465
Compliance with general behaviors	1.336	1.085	1.646	.006
Compliance with lifestyle score	1.057	.865	1.293	.587
Time since undergoing metabolic and bariatric surgery	.771	.592	1.005	.054
Pre-surgery BMI score	1.052	1.018	1.088	.003
Constant	.187			.146

*BMI*, body mass index; dependent variable, %TWL ≥ 35% of baseline weight post-MBS (no/yes)

**Fig. 4:**
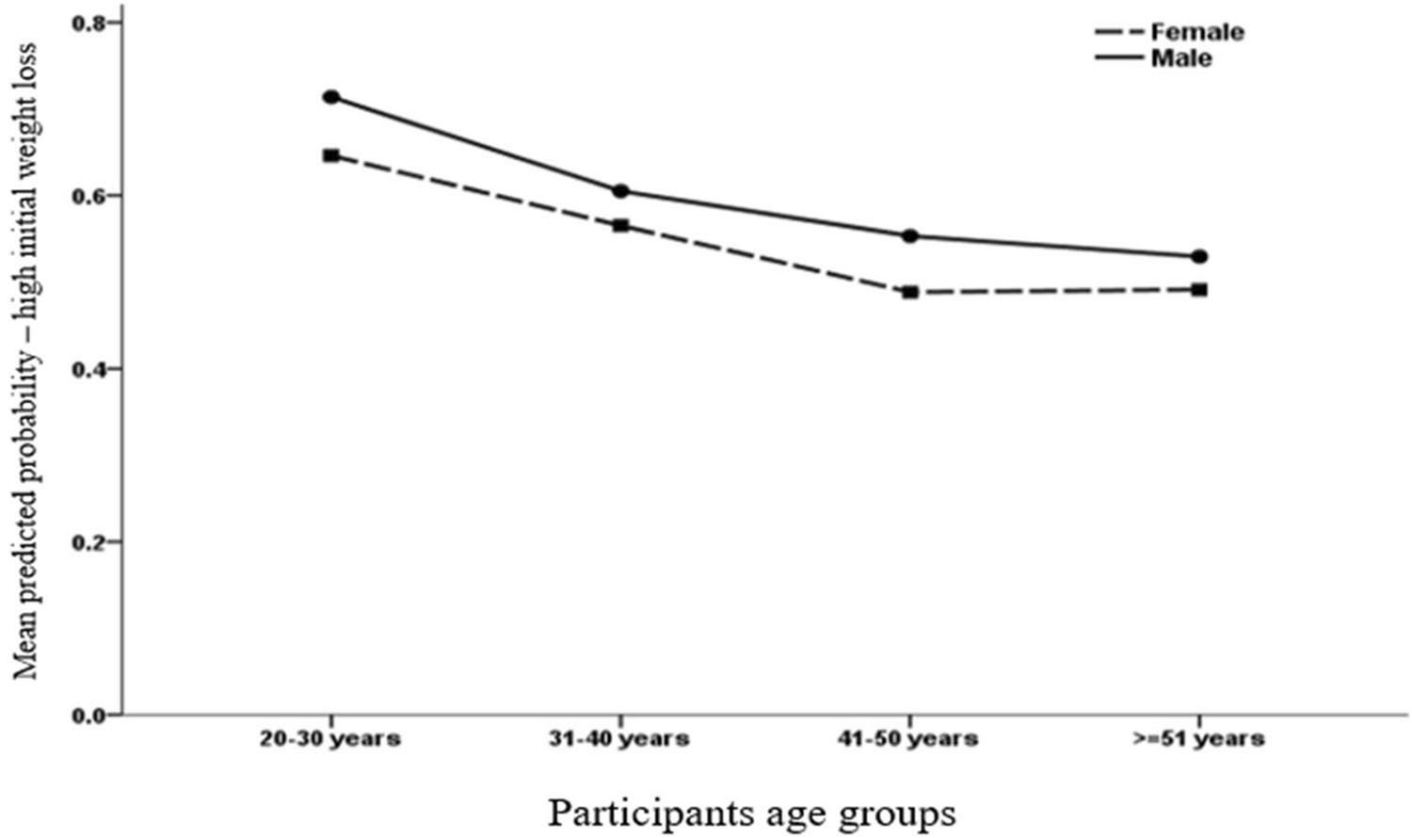
The association between participants’ age groups with their model mean predicted probability of %TWL post-surgery with separate lines for each sex

Figure 4 indicates that regardless of gender, participants’ chances of achieving significant weight loss decreased as they aged. Males appear to have higher odds of losing 35% or more of their baseline weight in the post-surgical period than females in all age groups.

#### % Weight Loss Maintenance

Table 7 revealed that 58.7% of the participants maintained > 85% of their weight loss post-surgery. Table 8 indicates that younger participants were more likely to maintain weight loss post-surgery, with *P* < 0.036. Participant compliance with food consumption behaviors did not correlate with %WLM, *P* < 0.853. However, participants were 22 times more likely to maintain weight loss when complying with drinking behaviors and 40 times more when complying with lifestyle behaviors.

**Table 7:** %WLM that is maintained > mean of 84.1%

	Frequency (%)
Low	161 (41.3)
High	229 (58.7)

**Table 8:** Multivariate logistic binary regression analysis of bariatric participants on %WLM over time

	Adjusted odds ratio (OR)	95% C.I		*P*-value
		Lower	Upper	
Age (years)	.974	.951	.998	.036
Sex = male	1.264	.798	2.003	.318
Educational level	.810	.632	1.037	.094
Compliance with food consumption score	1.018	.840	1.234	.853
Compliance with drink consumption score	1.228	1.019	1.479	.031
Compliance with general behaviors	1.181	.970	1.438	.098
Compliance with lifestyle score	1.402	1.152	1.706	.001
Time since undergoing metabolic and bariatric surgery	.658	.511	.847	.001
Pre-surgery BMI score	1.030	.998	1.063	.068
Constant	.626			.670

*BMI*, body mass index; dependent variable, high %WLM (> 84%) of loss weight (no/yes)

#### Weight Regain (WR)

WR mean scores of 8.34 kg were used to categorize participants with low or high WR.

Table 9 reveals that 39.2% of the participants regained 8.34 kg or more of their nadir weight. Table 10 indicates that the participants’ gender was correlated with their WR; males were three times more likely than females to regain weight post-MBS (*P* < 0.001). Furthermore, younger participants were more likely to regain weight post-surgery, with *P* < 0.016.

**Table 9:** WR from nadir weight > 8.34

	Frequency (%)
Weight regain < 8.34 kg	237 (60.8)
Weight regain ≥ 8.34 kg	153 (39.2)

**Table 10:** Multivariate logistic binary regression analysis of bariatric participants’ WR

	Adjusted odds ratio (OR)	95% C.I		*P*-value
		Lower	Upper	
Sex = male	2.820	1.651	4.816	< 0.001
Age (years)	.966	.939	.994	.016
Time since undergoing metabolic and bariatric surgery	1.586	1.194	2.107	.001
Pre-surgery BMI score	1.088	1.048	1.129	< 0.001
Post-surgery %TWL score	.896	.869	.923	< 0.001
Compliance with food consumption score	1.038	.837	1.288	.734
Compliance with drink consumption score	.837	.681	1.029	.091
Compliance with general behaviors	1.096	.876	1.372	.422
Compliance with lifestyle score	.721	.580	.896	.003
Constant	1.143			.905

*BMI*, body mass index; dependent variable, high WR (8.34 kg) of nadir weight (no/yes)

Compliance with food and general behaviors scores did not correlate with the WR following bariatric surgery. However, participants who complied with the lifestyle behaviors were less likely to regain weight (*P* < 0.003).

## Discussion

Patient compliance with recommendations is key to the success of any intervention [32]. Adherence to dietary and lifestyle guidelines and clinical follow-up after MBS can influence long-term weight outcomes [33]. WR is commonly associated with behavioral factors such as poor eating habits and lack of exercise [34, 35]. It is critical for patients to follow post-surgical dietary instructions to minimize nutrition-related complications and post-operative weight gain [36, 37].

To the best of our knowledge, this is the first study to translate and validate the EBBS questionnaire for the Arabic population. To accomplish this, we translated and validated the EBBS questionnaire using the Modern Standard Arabic to ensure better understanding by the general population of Arabic speakers.

In our results, we validated the translated version of the EBBS after excluding question 5 for several reasons. After receiving responses from the experts in content validity examination and calculating the I-CVI and S-CVI, we found that the CVI values of all questions exhibited high agreement except question five. This question had a low CVI score; some content experts believed that the item “How many times a week do you drink wine or alcohol?” interfered with the Islamic religion, as alcohol use is forbidden in Islamic culture and is seldom consumed. Furthermore, when the statistical properties of the items were considered, question 5 did not appear to function more effectively, at least for our sample; thus, we omitted the item to increase the validity and internal reliability of the questionnaire.

By applying the EFA/PCA, we found that the 10 indicators did not result in meaningful, simple, and theoretically sound latent factors; all the items loaded differently from their intended latent factors. Moreover, the UniCo, M-IREAL, and ECV indicated that the 10 indicators of the EBBS questionnaire might not necessarily comprise a unidimensional trait.

These closeness-to-unidimensionality tests are favorable for scale unidimensionality when the UniCo value is above 0.95 and the ECV value is greater than 0.85 [26]. This standard was also supported by the Cassilith-scree figure and PA test, which showed the presence of three to four latent factors with item-factor. However, the item-factor loadings obtained from the EFA did not correspond to the authors’ proposed structure because the EBBS questionnaire characterized the 11 traits as single-measured latent factors intended to measure various aspects applicable to post-MBS patients. Thus, the current study treated the scale as devised by its first authors [17] by computing item parcels and treating the 11 indicators as single-measured latent factors. However, the psychometric properties of the translated tool demonstrated an improvement in overall internal consistency (0.851) compared to the original questionnaire (0.743).

We classified an increase of 8.34 kg or more from nadir weight as high WR; our study demonstrated that males had higher WR than females after MBS. The same result was found in Afshan Masood et al. [38], in which males were found to be more likely to regain weight post-surgery than females, with approximately 15.3 ± 10.9 kg in males. In contrast, the retrospective cohort study demonstrated that gender is not a predictor of WR after MBS. This variation could be explained by a different definition of WR [39].

We calculated Pearson’s correlations between the bariatric patients’ eating behavior scores and their weight-related outcomes in the four domains. According to our findings, participants who followed food (EBBS domain A), general behavior (EBBS domain C), and lifestyle (EBBS domain S) recommendations by exercising and strictly monitoring their body weight lost more weight after surgery. Conversely, participants’ adherence to drinking habits (EBBS domain B) had no effect on weight loss after surgery. This finding was similar to a meta-analysis that included 14 studies and 19 reviews; physical activity was associated with higher weight loss post-MBS [40]. Moreover, a cross-sectional study found that physical activity was the only post-surgical behavior associated with weight loss at follow-up, in contrast to eating and drinking behaviors [41].

Additionally, participants who adhered to lifestyle behaviors (EBBS domain S), drink consumption (EBBS domain B), and general behavior (EBBS domain C) were more likely to maintain their weight. The same result was found in the original study on the impact of lifestyle behaviors besides affecting beverage consumption to maintain weight [17]. Another cohort study supported our finding by indicating a strong association between lifestyle behaviors and weight loss maintenance [38]. Interestingly, these three scales not only affect weight maintenance but also protect against WR. Studies of WR indicate that excessive beverage consumption [42] with a lack of adherence to exercise may contribute to WR [35, 43]. These findings illustrate that adherence to lifestyle recommendations (i.e., regular exercise and frequent weight measuring) is related to weight control.

### Strengths and Limitations

This is the first study to translate and validate the EBBS questionnaire for the Arab population. No other studies have examined the scale’s psychometric properties as they were used in this study. Furthermore, we included a large sample size. In our study, gender distribution was nearly equal. However, there were some limitations, including the fact that the study was conducted as a self-reported online survey, which may risk selection or information bias. Our inclusion criteria were generalized for all types of MBS; we were unable to compare the outcomes of different types of surgeries since we could not access the medical records of participants.

### Conclusions

The validation and reliability of the Arabic-language version of the 10-item, 4-subscale EBBS questionnaire were satisfactory. The study findings revealed an association between weight-related outcomes and the EBBS scales; however, more research into these associations is necessary. The presence of an Arabic version of the questionnaire is useful for estimating the level of patient adherence to dietary and lifestyle recommendations and recognizing factors that influence the efficacy of MBS. We suggest applying the instrument in a clinical setting to investigate the effect of different types of MBS on eating behavior outcomes.

## Supplementary Information

Below is the link to the electronic supplementary material.

Supplementary file1 (DOCX 20 KB)

## Data Availability

The datasets used and/or analysed during the current study are available from the corresponding author on reasonable request.
