# Supplementary material for: Translation and Validation of the Arabic Version of the Eating Behavior After Bariatric Surgery (EBBS) Questionnaire
Source: Obes Surg. 2023 Feb 13;33(4):1108–20. doi: 10.1007/s11695-023-06480-y (PMC10079758; doi:10.1007/s11695-023-06480-y)
Supplement: Supplementary file 1 — Supplementary file1 (DOCX 20 KB) [file 11695_2023_6480_MOESM1_ESM.docx]

**Supplementary Material**

**Supplementary**:

## Supplementary 1.The relevance ratings on the item scale by ten experts

|  | Expert 1 | Expert 2 | Expert 3 | Expert 4 | Expert 5 | Expert 6 | Expert 7 | Expert 8 | Expert 9 | Expert 10 |  | Expert in agreements | I-CVI | UA |
| --- | --- | --- | --- | --- | --- | --- | --- | --- | --- | --- | --- | --- | --- | --- |
| Item |  |  |  |  |  |  |  |  |  |  |  |  |  |  |
| Q 1 | 1 | 1 | 1 | 1 | 1 | 1 | 1 | 1 | 1 | 1 |  | 10 | 1 | 1 |
| Q 2 | 1 | 1 | 1 | 1 | 1 | 1 | 1 | 1 | 1 | 1 |  | 10 | 1 | 1 |
| Q 3 | 1 | 1 | 1 | 1 | 1 | 1 | 1 | 1 | 1 | 1 |  | 10 | 1 | 1 |
| Q 4 | 1 | 1 | 1 | 1 | 1 | 1 | 0 | 1 | 1 | 1 |  | 9 | 0.9 | 0 |
| Q 5 | 1 | 1 | 0 | 1 | 1 | 0 | 1 | 0 | 1 | 0 |  | 6 | 0.6 | 0 |
| Q 6 | 1 | 1 | 1 | 1 | 1 | 1 | 1 | 1 | 1 | 1 |  | 10 | 1 | 1 |
| Q 7 | 1 | 1 | 1 | 0 | 1 | 1 | 1 | 1 | 1 | 1 |  | 9 | 0.9 | 0 |
| Q 8 | 1 | 1 | 1 | 0 | 1 | 1 | 1 | 1 | 1 | 1 |  | 9 | 0.9 | 0 |
| Q 9 | 1 | 1 | 1 | 1 | 1 | 1 | 1 | 1 | 1 | 1 |  | 10 | 1 | 1 |
| Q10 | 1 | 1 | 1 | 1 | 1 | 1 | 1 | 1 | 1 | 1 |  | 10 | 1 | 1 |
| Q11 | 1 | 1 | 1 | 1 | 1 | 1 | 1 | 1 | 1 | 1 |  | 10 | 1 | 1 |
|  |  |  |  |  |  |  |  |  |  |  |  | S-CVI/AVE | 0.94 |  |
| Proportion relevance | 1 | 1 | 0.91 | 0.82 | 1 | 0.91 | 0.91 | 0.91 | 1 | 0.91 |  | S-CVI/UA |  | 0.63 |
|  |  |  |  |  |  |  |  |  |  | Average proportion of items judged as relevance across the ten experts | 0.94 |  |  |  |

Note: I-CVI = item content validity index; UA = universal agreement; S-CVI = scale content validity index; S-CVI/AVE = scale-level content validity index based on the average method; S-CVI/ UA = scale-level content validity index based on the universal agreement method.
